# VYC‐17.5L is Safe and Effective in Improving Skin Quality and Volume Deficit of Hands: Results From an 18‐Month Open‐Label Study

**DOI:** 10.1111/jocd.70675

**Published:** 2026-02-15

**Authors:** Daniela Greiner‐Krüger, Christophe Leys, Sylwia Lipko‐Godlewska, Graeme M. Kerson, Smita Chawla, Carola de la Guardia

**Affiliations:** ^1^ MediCorium, Zentrum Für Dermatologie Und Ästhetik Oberursel Germany; ^2^ Medical Skincare Sint‐Truiden Belgium; ^3^ Sylwia Lipko‐Godlewska, Praktyka Lekarska Krakow Poland; ^4^ Allergan Aesthetics, an AbbVie company Marlow UK; ^5^ Allergan Aesthetics, an AbbVie company Irvine California USA; ^6^ Allergan Aesthetics, an AbbVie company Madrid Spain

**Keywords:** dermal fillers, hand rejuvenation, hyaluronic acid, skin aging, volumization

## Abstract

**Background:**

Hands undergo age‐related changes in volume and skin quality. This 18‐month exploratory study evaluated safety and effectiveness of VYC‐17.5 L for hand rejuvenation.

**Methods:**

Participants (≥ 35 years) with moderate‐to‐extreme Allergan Hand Volume Deficit Scale (AHVDS) scores received VYC‐17.5L in both hands on day 1, with optional touch‐up on day 30. Effectiveness was assessed by AHVDS responder rate (≥ 1‐grade improvement from baseline per hand) at months 1, 3 (primary effectiveness timepoint), and at 3‐month intervals through month 18; investigator‐ and participant‐Global Aesthetic Improvement Scale (GAIS); hand Self‐Perception of Age (SPA); and skin quality measurements. Safety was evaluated throughout.

**Results:**

Seventy‐five participants (94.7% female; mean age, 53.89 years) had both hands injected with VYC‐17.5L (median, 2.0 mL/hand), and 60.0% of hands received touch‐up (median, 1.0 mL/hand). The primary endpoint was met: 100.0% of hands were AHVDS responders at month 3. The 100% response rate was maintained through month 6, and the majority of participants (> 65%) were responders through month 18. Most hands (≥ 85%) improved on investigator‐ and participant‐GAIS through month 12. SPA improved by 5 to 6 years. Significant skin quality improvements persisted to 12 months. Twenty‐two (29.3%) participants experienced treatment‐related adverse events; most were mild or moderate in severity with a mean duration of 45.1 days, and none led to study discontinuation.

**Conclusion:**

VYC‐17.5L is an effective treatment for improving hand volume deficit, with aesthetic benefits lasting 18 months.

## 1 Introduction

The hands are a prominently visible feature that undergo characteristic changes with age. These changes include subcutaneous volume loss and thinning of the dermis that lead to visible protrusion of veins, tendons, and the metacarpal bones on the dorsum of the hands [1]. Volume loss and changes in the skin quality of the hands can also occur in populations of all ages due to various factors (e.g., low body weight, excessive sun exposure). Compared to facial treatments, rejuvenating hands can be more challenging due to intricate, complex, and delicate anatomy, as well as how mobile, frequently used, and exposed they are. The 19 bones that make up each hand are covered by thick, glabrous skin on the palmar surface and thin, pliable skin on the dorsal surface. Within the subcutaneous tissue of the dorsum, 3 distinct fatty laminae (dorsal superficial lamina, dorsal intermediate lamina, and dorsal deep lamina) are separated by fascial layers that contain vasculature, cutaneous nerves, and tendons [1, 2, 3]. The superficial location of the rich vascular network and lymphatics make the dorsum delicate and prone to edema, which adds to the complexity of hand rejuvenation.

Despite the clearly visible signs of aging and changes in skin quality observed on the hands, there are only a few published studies about the use of soft tissue hyaluronic acid (HA) fillers for hand augmentation, and most have limited numbers of participants or limited follow up. VYC‐17.5L (Juvéderm Volift; Allergan Aesthetics, an AbbVie company, Irvine, CA, USA) is an HA soft tissue filler containing 17.5‐mg/mL HA and 0.3% w/w lidocaine indicated for the treatment of any deep skin depressions due to conditions such as premature aging. The safety and effectiveness of VYC‐17.5L in treating multiple areas (nasolabial folds, lips, radial cheek lines, forehead, and marionette lines) have been demonstrated in several clinical studies [4, 5, 6, 7, 8, 9, 10]. A recent prospective, single‐center study with 33 female participants showed that VYC‐17.5L treatment improved volume deficits of the hand and subjective improvement in the aesthetic appearance of the hands 6 months posttreatment, with durable results at 12 months posttreatment [8]. This study examined the safety and effectiveness of VYC‐17.5L for treating skin depressions of the hands with a larger study population and a longer study duration.

## 2 Methods

### 2.1 Study Design

This was an exploratory, prospective, single‐center, open‐label study conducted between May 2021 and December 2022 to evaluate the effectiveness and safety of VYC‐17.5L for treating skin depressions of aging hands. Three aesthetic dermatologists served as treating investigators (TIs). The study included 9 visits: screening, an initial treatment visit (plus optional touch‐up at month 1), and follow‐up visits at months 1, 3, 6, 9, 12, 15, and 18 posttreatment. The study planned to enroll 89 participants (178 hands) based on estimates from a previous pilot study using VYC‐17.5L in the hands. A sample size of 142 hands produces a 2‐sided 95% confidence interval (95% CI) with a width of 12.0%, assuming a conservative primary endpoint response rate of 85%. Assuming a 20% attrition rate, 89 participants (178 hands) were planned to be enrolled in this study. This study was conducted in accordance with the ethical principles outlined in the Declaration of Helsinki and all local regulatory requirements. All participants provided informed consent prior to inclusion.

### 2.2 Participants

Eligible participants were aged 35 years or older with both hands rated as moderate, severe, or extreme on the validated Allergan Hand Volume Deficit Scale (AHVDS) by the evaluating investigator (EI). To be eligible, participants had to agree not to change their normal hand care regimen or receive any other hand cosmetic treatments during the study; the use of their usual hand creams was allowed. Participants were not eligible if they had undergone any temporary hand dermal filler injections within 12 months before study entry; had previously undergone hand surgery, tissue grafting, or tissue augmentation with any permanent or semi‐permanent dermal fillers; had a history of hypertrophic scarring on hands; or had any fibrosis, scarring, or deformities on the hands. Participants were also excluded if they were receiving or were planning to receive anticoagulant therapy, anti‐inflammatory drugs, or other substances known to increase coagulation time for 10 days prior to study treatment and 3 days after study treatment.

### 2.3 Study Intervention

VYC‐17.5L was injected into both hands by 1 of the 3 TIs on day 1 of the study, with an optional touch‐up injection on month 1 by the same TI. The TI determined the volume injected (not to exceed 3 mL per hand for both initial and touch‐up injections, with total not to exceed 6 mL), the use of needle or cannula, and injection technique based on their clinical experience. All injections were followed by massage of the area.

### 2.4 Effectiveness Endpoints

The primary effectiveness endpoint was the proportion of hands showing a ≥ 1‐point improvement in the AHVDS (AHVDS responder) from baseline to the 3‐month visit, as assessed live by the EI. Secondary effectiveness endpoints were assessed by the EI at months 3, 6, 9, 12, 15, and 18 and included AHVDS responder rate at all follow ups and investigator‐rated Global Aesthetic Improvement Scale (GAIS), where responder rate was defined as the proportion of hands rated as “improved” or “much improved.” Participant assessments included participant‐rated GAIS at months 3, 6, 9, 12, 15, and 18 and Self‐Perception of Age (SPA) of the hands. Hand SPA was evaluated at baseline and at all follow ups using a 3‐point questionnaire where participants estimated the visible age of their hands compared to their actual age. One questionnaire was completed for both hands.

Skin quality measurements were also assessed on 1 hand (chosen randomly) at baseline and at months 3, 6, 9, 12, 15, and 18, as follows.: Skin roughness was measured using 3D images with interference fringe projection, captured with the PRIMOS 3D Lite system (Canfield Scientific). The parameters measured were Ra (average roughness), Rz (average height of roughness), and Rt (maximum height of roughness). Skin hydration was measured with the MoistureMeterD instrument (Delfin Technologies) using the S15 and XS5 probes, which measure 1.5 mm (hypodermis) and 0.5 mm (superficial dermis) deep, respectively. Skin elasticity was measured using the Cutometer dual MPA 580 (Courage & Khazaka). Parameters analyzed were Ue, Uf, Uv, Ur, Ua, Ur/Ue, Uv/Ue, and Ua/Uf. The mean of 2 measurements on each zone was calculated for each parameter.

### 2.5 Safety Endpoints

The incidence, severity, and duration of injection site reactions (ISRs) were captured by participants for the 30 days after initial and touch‐up injections. Adverse events (AEs) were recorded by the EI at each follow‐up visit and described by preferred term and relationship to the product. The EI‐provided event descriptions could include additional detail beyond the preferred term (e.g., inflammatory reaction characterized by edema, redness, heat, itching). Hand function was measured at all follow‐up visits by the EI using a finger goniometer, hand dynamometer, and pinch gauge.

### 2.6 Statistical Analyses

The treated population included all participants who consented, enrolled in the study, and were treated; this population was used to assess safety and effectiveness. The evaluable analysis set (EAS), which was used for effectiveness, included participants who received VYC‐17.5L treatment, completed all follow ups, and had no major protocol violations. Demographic and baseline characteristics and injection parameters are summarized descriptively. For the primary effectiveness endpoint, AHVDS responder rate for each hand (≥ 1‐grade improvement from baseline to the 3‐month visit) and corresponding 97.5% CI are summarized; this analysis was also done by side (left and right) and for AHVDS responder rate for both hands. For AHVDS, GAIS, and hand SPA, evaluations on each hand were considered independent measurements. When applicable, a participant‐level analysis was performed based on the worse grade obtained between both hands. Post hoc analyses were performed to explore the effect of baseline characteristics and injection volume (initial + touch‐up) on effectiveness and safety outcomes. Safety data (i.e., ISRs, AEs) are summarized descriptively using the preferred term for event type, severity, duration, and relationship to product. All statistical tests were assessed at a *α* = 5% level of significance. Statistical analyses were completed with Microsoft Excel 2010 and SAS 9.4.

## 3 Results

### 3.1 Participants

A total of 75 participants were enrolled and received the study treatment, constituting the treated population. Seventy‐two participants completed the final month 18 visit. Two participants were lost to follow up, and 1 participant withdrew consent. The hands of 2 participants were excluded from evaluation at the primary endpoint; 1 participant's right hand was scored as mild on the AHVDS at baseline and did not meet inclusion criteria, and another participant's left hand could not be scored due to hand edema. The EAS population comprised 71 participants.

Participants in the treated population were mostly female (94.7%) and had a mean age of 53.89 years (range, 35–69 years). Fitzpatrick skin phototypes from I to IV were represented in the study (Table 1). At baseline, the majority of hands had an AHVDS score of moderate (56.7%, *n* = 85), followed by severe (30.7%, *n* = 46) and extreme (12.0%, *n* = 18).

**TABLE 1:** Participant demographics and baseline disposition.

	Treated population *N* = 75 participants
Sex, *n* (%)	
Female	71 (94.7)
Male	4 (5.3)
Age, years	
Median	54
Range	35–69
Fitzpatrick skin phototype, *n* (%)	
I	5 (6.7)
II	41 (54.7)
III	19 (25.3)
IV	10 (13.3)
Baseline AHVDS score, *n* (%)^a^	
0 – None	0 (0.0)
1 – Minimal	1 (0.7)
2 – Moderate	85 (56.7)
3 – Severe	46 (30.7)
4 – Extreme	18 (12.0)

Abbreviation: AHVDS, Allergan Hand Volume Deficit Scale.

^a^ Percentages calculated based on *N* = 150 hands.

### 3.2 Treatment Administration

One hundred and fifty hands were treated with VYC‐17.5L at baseline, and 90 hands (60.0%) received touch‐up treatment at month 1 (Table 2). The median injected volume for the initial treatment was 2.0 mL per hand (range, 0.9–2.0 mL) and for the touch‐up was 1.0 mL per hand (range, 0.3–2.0 mL), with a median total volume for both treatments of 2.43 mL per hand (range, 1.00–3.30 mL). All injections were subdermal. Most (98.9%) initial and touch‐up injections were performed with the fanning technique, except 1 follow‐up injection which used linear threading. All injections were performed with a cannula: 50% used a 25G × 38 mm cannula, 47.3% used a 25G × 50 mm cannula, and 1.3% used cannulas of both sizes.

**TABLE 2:** Treatment administration per hand.

	Initial injection (*N* = 150 hands)	Optional touch‐up (*n* = 90 hands)	Total (initial + touch‐up) (*N* = 150 hands)
VYC‐17.5L volume, mL			
Mean (SD)	1.76 (0.37)	1.04 (0.43)	2.39 (0.56)
Median	2.00	1.00	2.43
Injection technique, *n* (%)			
Fanning	150 (100.0)	89 (98.9)	
Linear threading	0 (0.0)	1 (1.1)	

*Note:* Note that all injections were subdermal and used a cannula.

Abbreviation: SD, standard deviation.

### 3.3 Effectiveness

**FIGURE 1:**
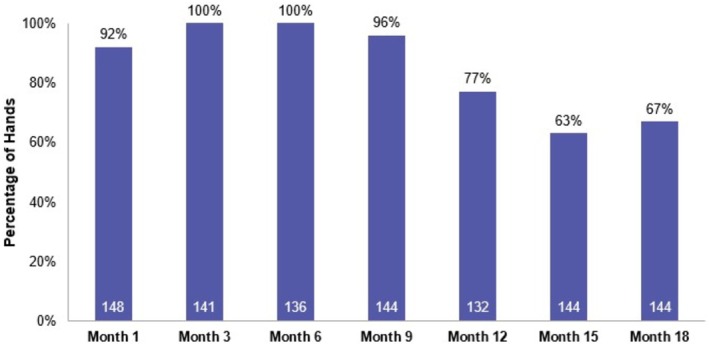
AHVDS responder rate. The proportion of hands in the treated population meeting AHVDS responder rate criteria, defined as a ≥ 1‐grade improvement from baseline. White text within bars indicates total number of hands evaluated. AHVDS, Allergan Hand Volume Deficit Scale.

The primary effectiveness endpoint was met: the EI‐assessed AHVDS responder rate among the treated population (*N* = 150 hands) at month 3 was 100% (97.5% CI [97.3%–100.0%]) for all evaluated hands. The 100% (97.5% CI [97.3%–100.0%]) AHVDS responder rate was maintained at month 6 and the majority of treated hands were responders through month 18 (month 9: 95.8%, 97.5% CI [91.2%–100.0%]; month 12: 77.3%, 97.5% CI [69.4%–100.0%]; month 18: 66.7%, 97.5% CI [58.6%–73.8%]) (Figure 1). Results were similar for the EAS population.

**FIGURE 2:**
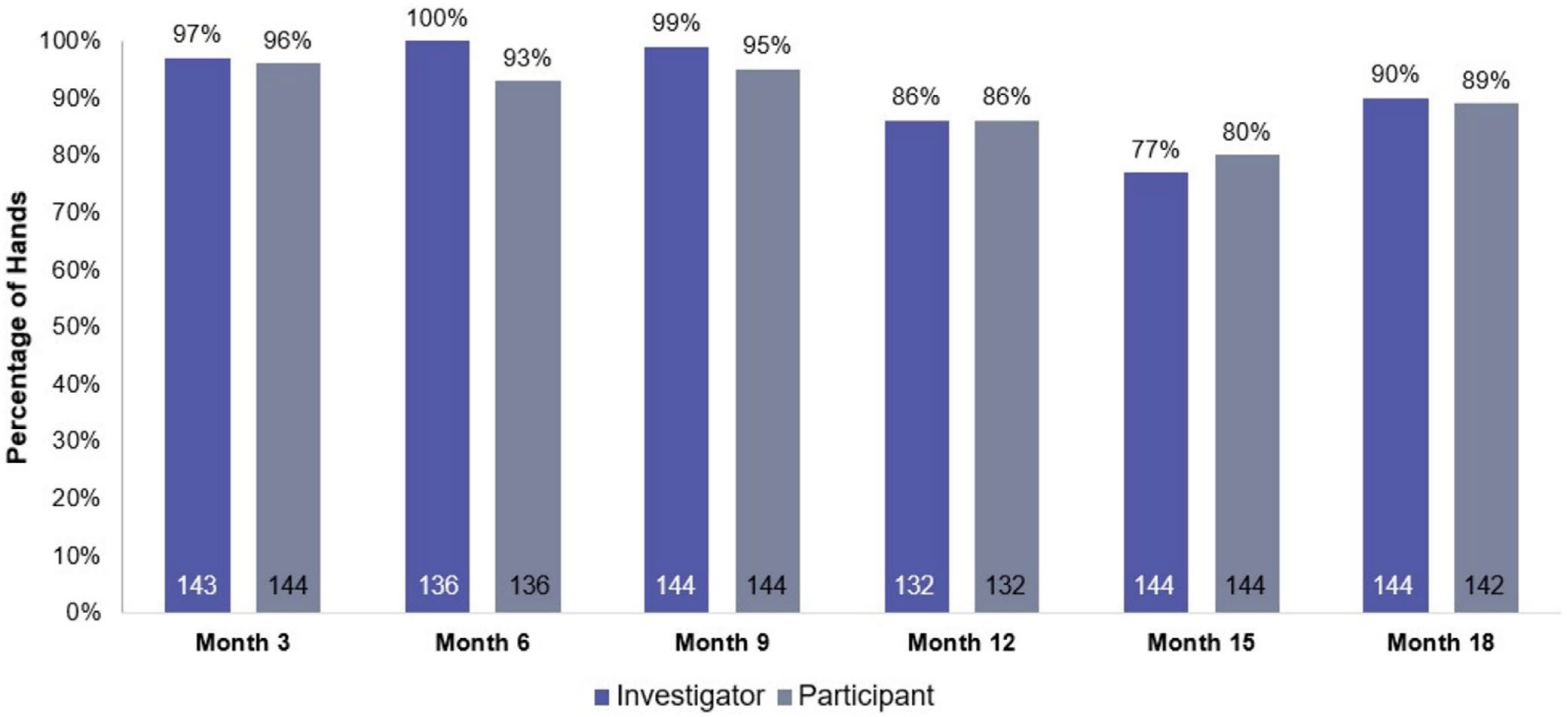
Investigator‐ and participant‐GAIS. Proportion of hands rated as “improved” or “much improved” compared to baseline by the evaluating investigator and participants. White text within bars indicates number of hands. GAIS, Global Aesthetic Improvement Scale.

At the primary effectiveness timepoint of 3 months posttreatment, the EI‐rated GAIS responder rate was high (97.2%; 97.5% CI [93.0%–100.0%]). Two hands were scored as worse due to edema present during the evaluation. Participant‐GAIS responder rates were also high (95.8%; 97.5% CI [91.2%–100.0%]) at month 3. The majority of hands were improved on both EI‐ and participant‐GAIS at all later timepoints posttreatment (Figure 2).

**FIGURE 3:**
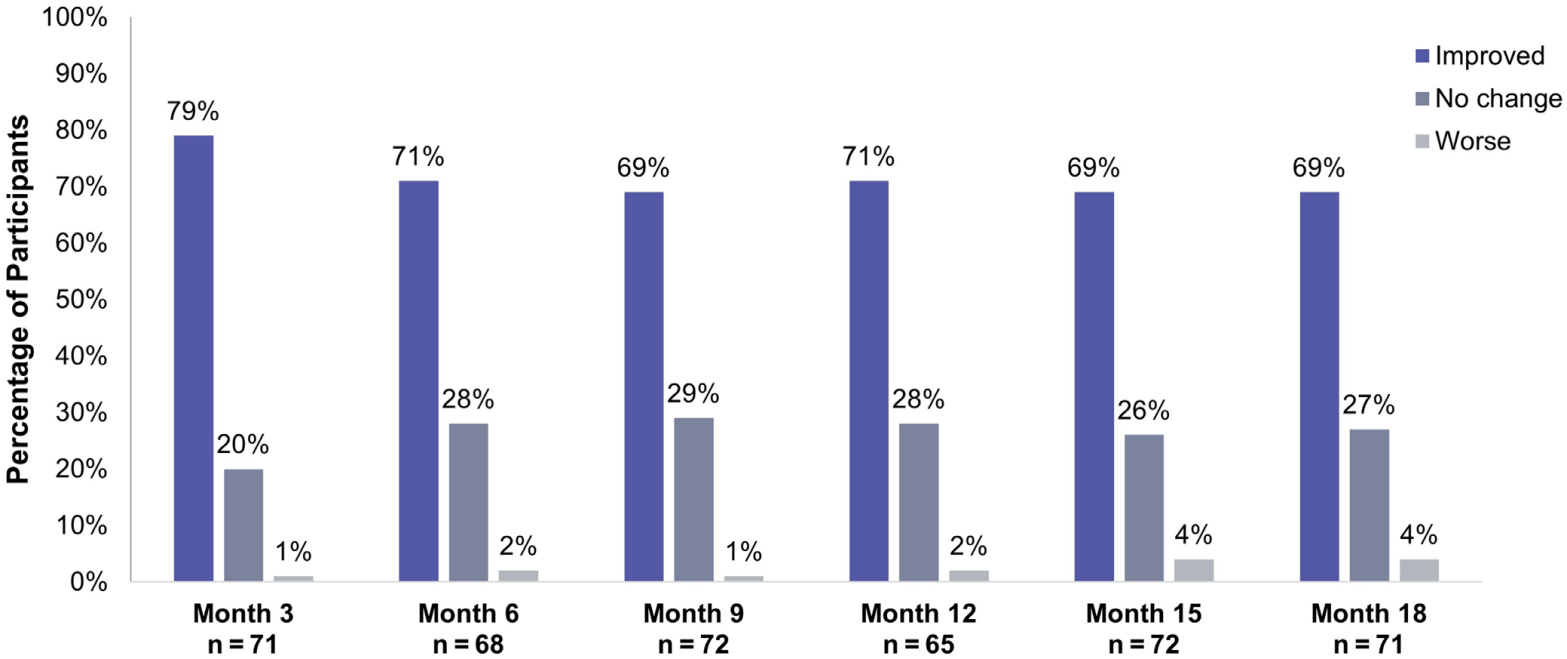
Hand self‐perception of age. Change from baseline in the perceived age of participants’ hands.

Prior to VYC‐17.5L treatment, 45% of participants rated their hands as looking older than their age. At all posttreatment timepoints, the majority of participants (~70%) reported improved perceived age of their hands. The mean self‐perceived improvement in the age of the hands was between 5 and 6 years younger than baseline age at all timepoints (Figure 3).

Post hoc analyses showed no significant correlation between the total volume of VYC 17.5L injected and clinical outcomes (i.e., the change from baseline in AHVDS score, GAIS, hand SPA) at any timepoint.

**FIGURE 4:**
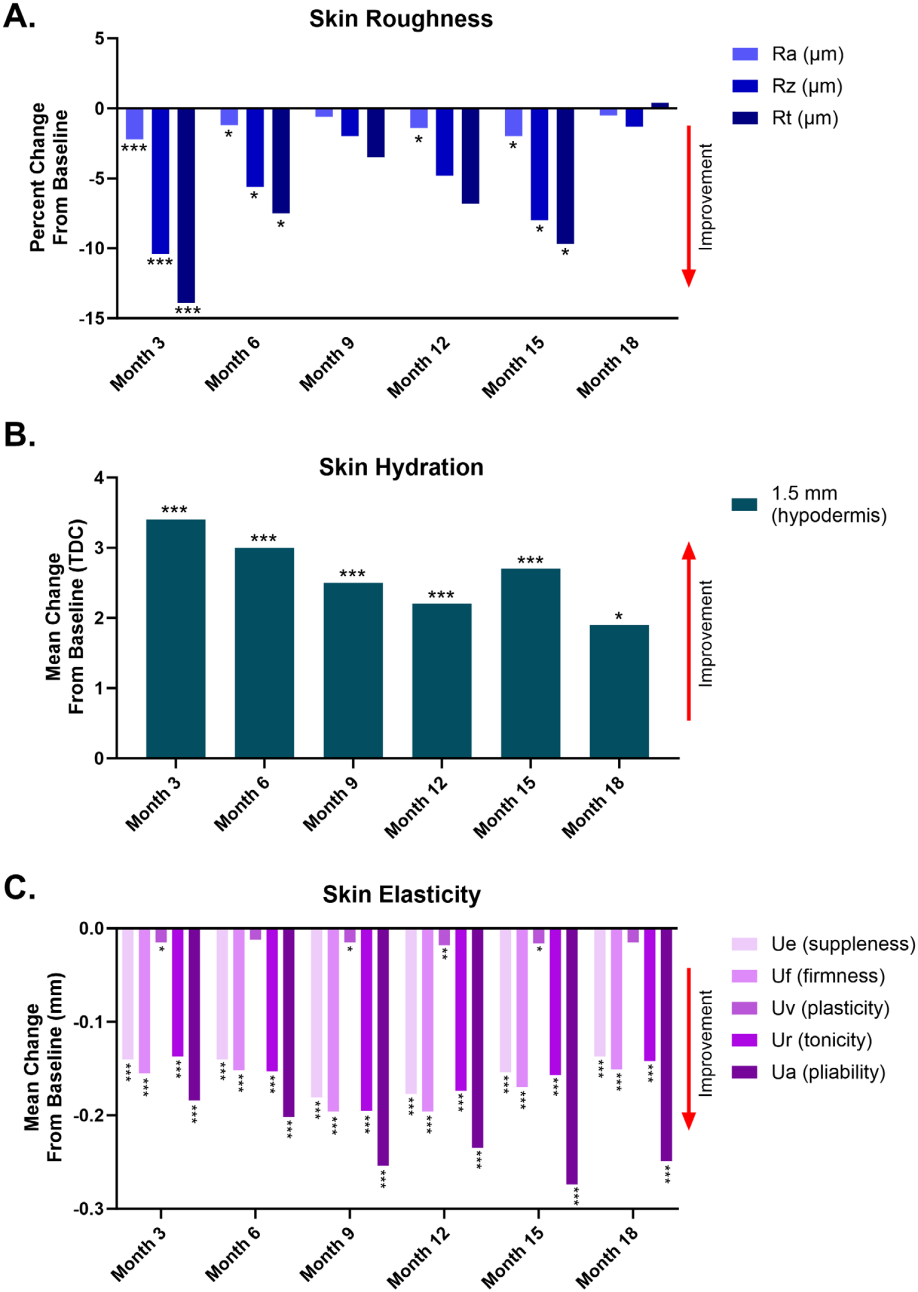
Changes in skin quality measurements after VYC‐17.5L. (A) Percent change from baseline in skin roughness parameters measured by Primos 3D. ***, *p* < 0.001; *, *p* < 0.05 vs. baseline. (B) Mean changes from baseline in hydration rate in the hypodermis, as measured by MoistureMeterD. Data are expressed in tissue dielectric constant (TDC). ***, *p* < 0.001; *, *p* < 0.05 vs. baseline. (C) Mean changes from baseline in skin elasticity parameters, as measured by Cutometer. ***, *p* < 0.001; **, *p* < 0.01; *, p < 0.05 vs. baseline.

At month 3, all skin roughness parameters had significantly decreased (with lower values indicating less roughness) from baseline (*p* < 0.0001). Significant improvements in roughness persisted through month 6 (*p* < 0.05) but were no longer significantly different from baseline at month 9, though the Ra parameter showed significant improvement at month 12. At month 15, all roughness parameters were significantly improved from baseline (*p* < 0.01) but were not significantly different from baseline levels by month 18 (Figure 4A). Skin hydration was significantly increased from baseline at months 3, 6, and 9 (*p* < 0.001); hydration levels at the level of the hypodermis (S15 probe measurements) were maintained through the month 18 follow up, though the significant improvements in hydration in the superficial dermis (XS5 probe) were no longer significantly different from baseline at the month 12 or month 18 follow ups (Figure 4B). In terms of skin elasticity, significant decreases from baseline in Ue, Uf, Uv (immediate, delayed and final extensibility) and Ur, Ua (immediate and total retractation) were observed at month 3, indicating firmer and less extensible skin (*p* < 0.05). Improvements in these parameters persisted through month 18, except for Uv, which was not statistically significantly different from baseline at months 6 or 18. A significant decrease of Ur/Ue and Ua/Uf and a significant increase of Uv/Ue compared to baseline were also observed at all follow‐up evaluations (*p* < 0.01). These changes characterize skin as firmer and less elastic (Figure 4C).

### 3.4 Safety

**FIGURE 5:**
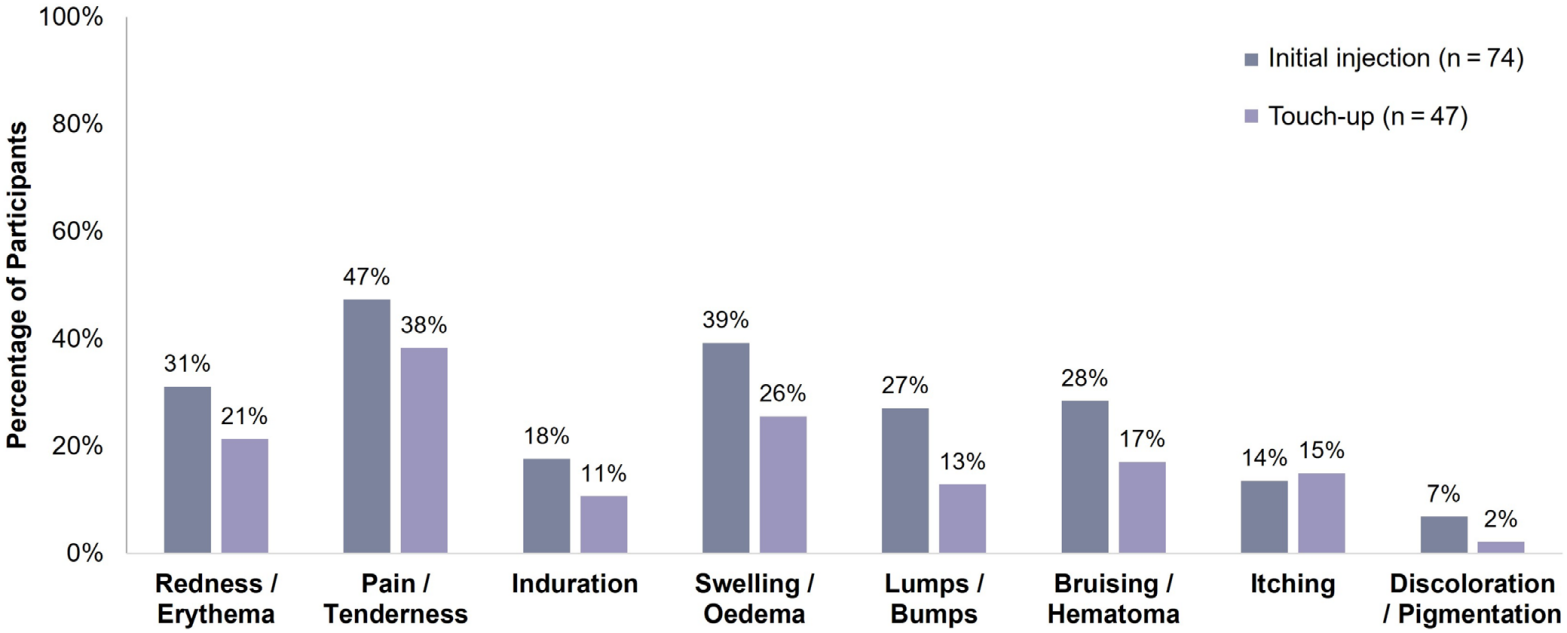
Injection site reactions. Proportion of participants with ISRs. ISR, injection site reaction.

After the initial injection, 68.9% (*n* = 51) of participants reported at least 1 ISR; the most commonly reported ISRs were pain/tenderness (47.3%), swelling/edema (39.2%), and redness/erythema (31.1%) (Figure 5). The majority of ISRs recorded after the initial injection were mild in severity and lasted between 1 and 3 days. Of the participants who received a touch up, 46.8% (*n* = 22) reported similar ISRs to those seen after initial injection. These ISRs were at slightly decreased rates, and the majority were mild in severity and resolved within 1 to 3 days. However, 1 participant reported ISRs of severe redness/erythema, swelling/edema, and lumps/bumps on both of their hands.

The incidence and severity of AEs were captured by the EI. There were no unexpected AEs. Forty‐nine participants (65.3%) experienced any AE during the study; 22 participants (29.3%) experienced at least 1 AE related to the product. The most common treatment‐related AEs were injection site inflammation (21.3%, *n* = 16 participants), injection site nodules/mass (9.3%, *n* = 7 participants), and injection site edema (5.3%, *n* = 4 participants). Most treatment‐related AEs were mild or moderate in severity; the only 2 severe treatment‐related AEs were edema (*n* = 1) and pain (*n* = 1). The mean duration of treatment‐related AEs was 45.14 days (range, 0–119 days) and 62% (*n* = 24) required treatment with oral medication (i.e., antibiotics, glucocorticoids, and/or antihistamines). All treatment‐related AEs resolved by the end of the study without sequela, and no serious AEs were observed.

Seven participants reported late‐onset nodules/masses, appearing ≥ 30 days after the last injection, and 14 participants presented with late‐onset inflammatory events that were characterized by edema, heat, redness, and itching. Three participants presented with late‐onset edema, 2 reported late‐onset pain, 2 presented with late‐onset discoloration, and 1 presented with late‐onset discomfort (Table 3). Potential triggering events (e.g., systemic infection, vaccination, local infection) were present in 6 participants who presented with late‐onset inflammatory events. The majority of late‐onset events were mild in severity. Thirteen participants presenting with late‐onset events received antibiotics, glucocorticoids, and antihistamines; 1 participant received antibiotics and glucocorticoids; 2 participants received a non‐opioid analgesic (doliprane/paracetamol/acetaminophen); and 4 were not prescribed oral medication. No participants were treated with hyaluronidase.

**TABLE 3:** Treatment‐related adverse events with onset ≥ 30‐days posttreatment.

Type of late‐onset event	Number of events	Number of participants^a^
Inflammatory	15	14
Nodule/mass	8	7
Edema	5	3
Discoloration	3	2
Pain	3	2
Discomfort	3	1

^a^ Five participants presented with 2 types of late‐onset events and 2 participants presented with 3 types of late‐onset events.

Post hoc analyses conducted to examine potential contributing factors for late‐onset AEs showed that the proportion of participants who experienced a late‐onset AE was higher (65%) among participants who received ≥ 5 mL total (initial plus touch up). Although the distribution of participants depending on total injection volume (≥ 5 mL versus < 5 mL) was not significantly different between groups with late‐onset events and those without late‐onset events, this is likely due to the small number of participants presenting with late‐onset AEs. Additional correlation analyses showed that the mean total injection volume was significantly higher in participants who experienced a late‐onset event than in participants who did not (*p* < 0.01). Additionally, although not statistically significant, an injector effect was observed for the appearance of late‐onset AEs, with ~60% of late‐onset AEs occurring in participants treated by one injector and with higher total injection volumes; this injector treated 30 participants (11 of whom presented with late‐onset events) with a mean total volume of 5.6 mL and a median total volume of 6.0 mL. There was no correlation between age, sex, or Fitzpatrick skin type and the occurrence of late‐onset AEs.

No clinically significant changes in hand function were observed except in 1 participant who experienced clinically significant loss of hand and finger strength (values = 0), as measured by hand dynamometer and pinch gauge, at month 3 due to edema of moderate severity on the dorsum of the hands. After touch‐up injection, the participant had reported ISRs of severe redness/erythema, swelling/edema, and lumps/bumps; the lumps/bumps and redness/erythema were transient (≤ 2 days) whereas the swelling/edema persisted for > 30 days and was therefore classified as an AE of inflammatory reaction deemed to have a causal relationship to the treatment. The participant received corrective treatment and the edema resolved by the month 6 follow‐up, at which time hand and finger strength returned to baseline values. The participant's medical history was unremarkable, but the injection volume (initial, 2.0 mL per hand; touch‐up, 1.0 mL per hand) may have factored in.

## 4 Discussion

This prospective, open‐label, single‐center study showed that all (100%) treated hands met the primary effectiveness endpoint, showing at least a 1‐grade improvement in AHVDS scores 3 months after initial treatment with VYC‐17.5L. A high responder rate was maintained through month 9, with the majority of participants remaining responders through the final study evaluation at month 18, supporting the effectiveness of VYC‐17.5L for improving volume deficit in hands. The primary effectiveness results, evaluated using the clinically validated AHVDS, were consistent with the high rates of GAIS improvement as assessed by both the EI and participants, which suggests that treatment with VYC‐17.5L in the hands was associated with meaningful and durable aesthetic improvements from the perspectives of both investigator and participant. Self‐perception of age of the hands by the participants was also improved, with 79% of participants finding an improvement in the perceived age of their hands at month 3% and 69% at month 18. The average improvement was 5 to 6 years at all time points posttreatment.

Treatment with VYC‐17.5L was also associated with an improvement in skin quality of the hands as assessed by objective, subjective, and biophysical measurements. Clinical assessments and participant‐reported outcomes were corroborated by improvements in objective measurements of skin quality, with significant improvements in roughness, hydration, and elasticity. The observed improvements in clinical probe measurements of skin quality and biomechanical properties add to the growing body of literature demonstrating that HA fillers positively influence multiple aspects of skin quality in the hands [11, 12]. More hydrated tissue may result from the hydrophilic HA increasing local water content and altering water transport within the skin [13, 14]. Treatment‐related changes in elasticity, where decreases in Cutometer‐measured elasticity represent a firmer and tighter skin that is less resistant to deformation, may be due to physical interactions between the HA gel and surrounding tissue affecting the composition and organization of extracellular matrix proteins (e.g., collagen, elastin) [14]. Fluctuations in skin roughness parameters over the course of the study (i.e., initial improvement followed by a slight dip around months 9–12 before improvement again around month 15) follow the approximate time course of filler degradation and increases in aquaporin that improve hydration levels leading to less roughness [14]. Additionally, the later study time points occurred during the summer months, which may have corresponded with seasonal improvements in skin quality compared to winter months where skin quality parameters showed less improvement compared with baseline.

ISRs reported in this study were of the type, severity, and duration expected of soft tissue HA fillers. No clinically significant impairment in hand function was observed other than a transient decline in strength due to edema in 1 participant at the month 3 visit; the impairment was resolved by the month 6 visit. There were no unexpected AEs and treatment‐related AEs reported in the study were consistent in nature with those expected based on prior studies of VYC‐17.5L in other anatomical areas as well as product labeling. No granulomatous reactions were reported. The type, severity, and duration of AEs observed in the present study are within the range of those reported in other studies of soft tissue filler treatment for aging hands [8, 11, 12, 15, 16, 17]. All treatment‐related AEs resolved by study end with routine treatment (i.e., antibiotics, glucocorticoids, antihistamines; if needed), without dissolution of filler using hyaluronidase, and without sequela. No participant discontinued due to an AE.

Factors that may have contributed to the occurrence of late‐onset AEs include the hands' delicate, intricate anatomy and their hyperdynamic nature. Due to the superficial location of blood and lymphatic vessels, hands are particularly prone to edema and inflammation. Posttreatment behavior, such as aggressive topicals or trauma, may predispose the appearance of late‐onset events in the hands. As a result, safety data (e.g., AEs, late‐onset events) on fillers for facial indications cannot be directly extrapolated to the hands. Indeed, in clinical trials of VYC‐17.5L for multiple facial indications (i.e., nasolabial folds, cheeks, lips, forehead, marionette lines, full face), late‐onset events were only reported in 2 of the 9 studies, with an incidence below 2.5% [4, 5, 6, 7, 8, 9, 18, 19, 20, 21, 22].

Injection volume may have also factored into the occurrence of late‐onset AEs. The TIs determined injection volumes based on their clinical discretion within the predefined limits of the study. Although in clinical practice, the TIs more commonly use ~1 mL per hand due to patient concerns with cost and/or sufficient correction, the TIs used higher volumes to better understand the potential impact of volume on duration of effect. Injection volume did not influence efficacy outcomes but did appear to affect safety, as participants who experienced late‐onset events received VYC‐17.5L in slightly larger volumes (~3 mL per hand) than the median volume used for the entire study population (2.43 mL per hand). Considering the touch‐up injection given at month 1, most participants (92% of hands) had already achieved a 1‐grade AHVDS improvement prior to touch up, and additional volume would have been unlikely to provide additional aesthetic benefits. This was supported by the post hoc analyses showing no differences in efficacy, but a higher incidence of late‐onset AEs, based on injection volume. There were no significant correlations between other factors (i.e., age, sex, Fitzpatrick skin phototype) and the incidence of late‐onset AEs.

Injection technique (i.e., targeting the superficial fascia using fanning, number and location of entry points, reach of the deposition of product, number of passes) was up to the discretion of each TI but was generally similar among all 3, including the use of cannula by all injectors. Although the post hoc data suggest that higher injection volume was a common factor for reported AEs, studies comparing needle versus cannula for filler treatment of the hands may be warranted, as there is not currently a consensus on the best injection instrument or technique for hand rejuvenation. The use of cannula versus needles may be associated with a potentially lower risk of vascular complications due to differences in the amount of force required to penetrate arterial walls [21, 23, 24]. Anatomical considerations also warrant direct comparison of injection instrument, as needles, for example, may require multiple entry points but can be targeted, whereas the use of a cannula may allow for fewer entry points and deposition in a specific layer, but depositing the filler using a fanning technique may disrupt the delicate structures of the dorsal hand leading to edema and/or swelling. There have been other reports with similar late‐onset AEs occurring > 3 weeks after soft tissue filler treatment of the hands [8, 15, 16, 25]. Although comparisons across studies are always difficult, findings from a recent study with an HA filler to correct volume deficit in hands reported late‐onset events in 28% of participants injected with a cannula, and the use of cannula was associated with an increased rate of TEAEs and alterations in thumb flexion compared to needle injections [15, 25].

Additionally, an injector effect was observed with ~60% of late‐onset events occurring in participants treated by one investigator. It is, however, difficult to identify more specific factors (e.g., differences in technique such as more or less sweeping movements with the cannula) that may have contributed to this effect and this investigator treated a greater proportion of participants (40%) than other injectors. It is also important to note that this study was conducted during the COVID‐19 pandemic. Participants may have been using alcohol‐based hand sanitizers and/or washing their hands at increased rates, which may have increased the sensitivity of skin in the treated areas and predisposed participants to AEs.

For safe administration of soft tissue fillers in the hands, it is important to have a thorough understanding of hand anatomy and injection technique [26, 27]. The utilization of ultrasound to guide injections may also provide a reduced incidence of AEs, but there are limited published data on ultrasound‐guided injection of filler into the hand [28, 29]. Also, as injected volume may be a factor, further research is needed to explore if lower injection volumes could lead to safer outcomes. Additionally, when looking at the published literature on hand volume augmentation using soft tissue fillers, larger studies with longer follow‐up tend to show increased rates of late‐onset events [8, 11, 12, 15, 16, 17]. Therefore, when designing future studies, it is important to include longer (> 12 month) observation periods and include a sample size that is sufficiently powered to detect these events over the study period.

Limitations of this study include the lack of a control arm, the single investigational site, the small number of injectors, no comparison of injection technique, and VYC‐17.5L injection by cannula only.

## 5 Conclusion

Treatment with VYC‐17.5L was associated with sustained improvements in the overall aesthetic appearance of the hands, as assessed by subjective and objective measurements. This study also suggests that, although late‐onset AEs were reported and may be explained by myriad factors, the overall safety profile of VYC‐17.5L for hand rejuvenation is within the range of other clinical data on hand rejuvenation using soft tissue fillers with no serious AEs reported [11, 15, 16, 17]. Aseptic technique, a thorough understanding of hand anatomy and injection technique, and thoughtful consideration of volume are important factors for the safe administration of soft tissue fillers in the hands. Overall, this study showed that treatment with VYC‐17.5L was highly effective for treating volume deficits and improving skin quality of hands for up to 18 months.

## Author Contributions

D. G.‐K., C. L., and S. L.‐G. contributed to study execution, data collection and interpretation, and writing – review and editing. G. M. K. and S. C. contributed to study oversight, data analysis and interpretation, writing – original draft preparation, and writing – review and editing. C. d. l. G. contributed to study design, study oversight, data analysis and interpretation, writing – original draft preparation, and writing – review and editing. All authors have read and approved the final manuscript.

## Funding

This study was supported by Allergan Aesthetics, an AbbVie company.

## Ethics Statement

The authors confirm that the ethical policies of the journal, as noted on the journal's author guidelines page, have been adhered to. This study was conducted in accordance with the ethical principles outlined in the Declaration of Helsinki and all local regulatory requirements, including approval of the protocol by an independent ethics committee. All participants provided informed consent prior to inclusion.

## Conflicts of Interest

The design, study conduct, and financial support for the study were provided by Allergan Aesthetics, an AbbVie company. AbbVie participated in the interpretation of data, review, and approval of the publication. Neither honoraria nor any other form of compensation was provided for authorship. Financial disclosures are as declared by authors. D. Greiner‐Krüger is an investigator, KOL, and speaker for Allergan Aesthetics, an AbbVie company. C. Leys is an investigator for Allergan Aesthetics, an AbbVie company. S. Lipko‐Godlewska is a speaker, investigator, and KOL for Allergan Aesthetics, an AbbVie company. G. M. Kerson, S. Chawla, and C. de le Guardia are employees of AbbVie Inc and may own company stock.

## Data Availability

AbbVie is committed to responsible data sharing regarding the clinical trials we sponsor. This includes access to anonymized, individual, and trial‐level data (analysis data sets), as well as other information (eg, protocols, clinical study reports, or analysis plans), as long as the trials are not part of an ongoing or planned regulatory submission. This includes requests for clinical trial data for unlicensed products and indications. These clinical trial data can be requested by any qualified researchers who engage in rigorous, independent, scientific research, and will be provided following review and approval of a research proposal, Statistical Analysis Plan (SAP), and execution of a Data Sharing Agreement (DSA). Data requests can be submitted at any time after approval in the US and Europe and after acceptance of this manuscript for publication. The data will be accessible for 12 months, with possible extensions considered. For more information on the process or to submit a request, visit the following link: https://vivli.org/ourmember/abbvie/ then select “Home”.
